# First-Hand Experiences of Autistic Students About Teacher Autonomy Support, Structure, and Involvement: A Video-Stimulated Recall (Interview) Study

**DOI:** 10.1007/s10803-025-06861-5

**Published:** 2025-05-08

**Authors:** Fernanda Esqueda Villegas, Steffie van der Steen, Alexander Minnaert

**Affiliations:** Department of Inclusive and Special Needs Education, Faculty of Behavioural and Social Sciences, University of Groningen, Grote Kruisstraat 2/1, Groningen, 9712 TS the Netherlands

**Keywords:** Inclusive education, Autistic students, Secondary education, Adolescents, Self-determination theory, Netherlands, Mexico

## Abstract

Opportunities for children and adolescents to share their views in society and research remain scarce, especially in terms of their first-hand learning experiences. This problem extends to autism research, where the voices of autistic people are under-represented. Therefore, this study investigated the classroom experiences of autistic students in mainstream secondary schools in the Netherlands and Mexico, focusing on their perceptions of teachers’ autonomy-support, structure, involvement and classroom interactions. Using video-stimulated recall (VSR) interviews, we recorded 13 students’ thoughts and emotional reactions while viewing two of their videotaped lessons. The data were analyzed qualitatively using both deductive and inductive approaches to provide a narrative of the aspects that autistic students reported affected their learning. Autistic learners had varied reactions to autonomy support, but they all valued different types of structure provided by their teachers, which increased their confidence in achieving goals (i.e. completing a task) and provided a sense of mastery. In particular, fear of making mistakes, especially due to negative reactions from classmates, was a recurring problem for autistic students. Teachers who were approachable, understanding and supportive made a significant difference for these students. Finally, autistic participants relied on both the teacher and peer interactions to gain clarity on tasks and move forward. Our findings highlight fundamental issues that all secondary school teachers could consider in their daily practice. We propose that VSR method can serve as a solid basis for conducting interviews with autistic youth and move towards a more inclusive approach in autism research.

The Convention on the Rights of the Child recognizes that children (defined as people under the age of 18) have the right to voice their perspectives on issues that concern them (UNICEF, 1989). Despite a worldwide adoption of this principle, opportunities for children and adolescents to share their views in society and research remain scarce (Alves et al., 2022), particularly regarding their first-hand learning experiences (Morgan, 2007). This issue extends to the field of autism research, where a substantial number of investigations have focused on the causes of autism. Yet, this area is not considered a main priority by autistic people or the broader autistic community (AARC, 2021; Cage et al., 2024; den Houting & Pellicano, 2019; Pellicano et al., 2014; Putnam et al., 2023). Since many autistic students are being enrolled in mainstream education (Ravet, 2018), research that aims to identify how these settings can be made more inclusive is regarded as a higher priority (Cage et al., 2024; Pellicano et al., 2014). In this line, autistic students often associate mainstream schools with negative emotions, such as feeling “unwanted” or “excluded” in them (Goodall, 2019). Indeed, many parents report that their autistic children struggle in mainstream environments, partly due to the lack of identification and understanding from schools and teachers regarding their needs (Brede et al., 2017; Cridland et al., 2014; Croydon et al., 2019; Makin et al., 2017). Identifying these needs, however, can be challenging for teachers, as autistic students are a heterogenous group whose strengths and weaknesses vary widely from one student to another (Anderson et al., 2024; Saggers et al., 2019). According to the DSM-5, autistic individuals’ support needs range from “requiring support” to “requiring very substantial support” (American Psychiatric Association, 2022). In practice, this means teachers will come across autistic students who have (above) average intellectual and language skills (Jarman & Rayner, 2015; Ravet, 2015) but also students who require a more tailored approach (Brede et al., 2017; Croydon et al., 2019). Despite their diverse profiles, all autistic learners experience social and communication difficulties (American Psychiatric Association, 2022), which can influence their inclusive education (Salceanu, 2020).

Evidence suggests that, while many teachers support the principle of including autistic students in mainstream education (Agyapong et al., 2010; Cassimos et al., 2015; Memisevic et al., 2024), some lack an understanding of autism (Anderson et al., 2024; McKinlay et al., 2022), doubt their ability to offer adequate support (Cassimos et al., 2015), or require further guidance on their teaching strategies (Ravet, 2018; Van Der Steen et al., 2020). For example, a group of Dutch educators in the study by Van Der Steen et al. (2020) stressed that knowing how to adapt their instructions to meet autistic students’ needs, as well as receiving suggestions of suitable materials, would benefit their practice. Similarly, Mexican teachers in a recent study expressed a desire to provide more structure and reduce the chaos in the classroom for their autistic students (Esqueda Villegas et al., submitted). Since some autistic students tend to be less engaged in the classroom setting (Roorda et al., 2021; Zajic et al., 2020), expanding teachers’ knowledge of effective approaches may be pivotal to supporting their learning experiences. In this sense, Self-Determination Theory (SDT) highlights teaching practices that should lead to students becoming motivated and engaged with the environment (Ryan & Deci, 2018).

Applied to the educational context, SDT proposes that *all* students have the need for autonomy, competence, and relatedness (Ryan & Deci, 2018). Teachers can support these needs by providing autonomy support, structure, and involvement (Snikkers-Mommer et al., 2024). Being autonomy supportive implies that teachers give students the freedom to make their own choices (Jang et al., 2010; Reeve & Jang, 2006). Providing structure can involve guidance during tasks, stipulating clear goal expectations and giving information on what the student did well or wrong in order to move forward (Jang et al., 2010; Minnaert, 2013; Skinner & Belmont, 1993). Showing involvement entails teachers being supportive and ensuring that students’ questions are acknowledged and answered (Ryan & Deci, 2018). In relation to SDT and autistic students, Heyworth et al. (2021) found that schools often impose classroom tasks on autistic learners rather than encouraging decision-making, thereby possibly thwarting their autonomy. Unsurprisingly, low levels of autonomy have been reported among these students (Chou et al., 2017). In terms of structure, step-by-step guidance can support autistic students’ goal attainment and prevent distressing emotions such as panic or stress (Anderson et al., 2024), enhancing their feelings of competence. Parents emphasize that explicit instructions, visual supports, and structured routines facilitate autistic children’s inclusion in mainstream education (Stephenson et al., 2021). Teacher involvement is similarly important, as educators who actively listen to autistic students and check in on their well-being can positively impact their school experiences (Saggers et al., 2011; Sciutto et al., 2012).

As autistic students’ progress through their school trajectories, difficulties in navigating the environment seem to increase (Brede et al., 2017; Carrington et al., 2003; Croydon et al., 2019; Hasson et al., 2024). In line with this, one autistic female in the study of Cridland et al. (2014) emphasized that secondary education was “much harder” than primary school. Even though previous research suggests that secondary schools present more social and academic challenges for autistic students (Humphrey & Lewis, 2008), only a handful of investigations have been conducted in this setting (Hill, 2014; Saggers, 2015). Additionally, the voices of autistic people have been under-researched (Costley et al., 2021; Goodall & MacKenzie, 2019; Hill, 2014). Instead, stakeholders such as parents or teachers often provide insights into the school experiences of autistic students (Goodall, 2018). While such stakeholders can offer valuable information, there may be a mismatch between *perceived* and *actual* needs of these students (Esqueda Villegas et al., 2024), which can only be accurately identified and understood by directly consulting autistic people (Haar et al., 2024). However, according to Lewis (2009), several issues might prevent autistic people’s voices from being adequately represented in research, such as the communication demands of certain techniques. For example, some autistic participants have difficulties to engage in interviews because they feel “too anxious” about the process and ultimately opt-out of participating (Anderson et al., 2024; Harrington et al., 2013; MacLeod et al., 2014). Lewis (2009) further adds that autistic children may be “more inclined to silence” or “I don’t know” responses compared to their peers. These challenges underscore the need for more inclusive research designs that accommodate the individual needs of autistic people and effectively capture their first-hand experiences (Autism CRC, 2016).

To contribute to the aforementioned area of research, we present the recalled experiences (from two videotaped lessons) of 13 autistic students enrolled in mainstream secondary schools in the Netherlands and Mexico; two United Nations member states with an inclusive approach to education. In both countries, secondary education is compulsory and students with special educational needs (SEN) are encouraged to attend mainstream settings (Diario Oficial de la Federación, 2011; Gubbels et al., 2018). Unfortunately, no prevalence data are available on autistic adolescents in these contexts. However, among children aged 12 or younger, prevalence estimates are 1% in the Netherlands (van der Gaag, 2018) and 0.87% in Mexico (Fombonne et al., 2016). Consistent with international literature, research on autistic students in secondary education remains limited in both countries (Hill, 2014), making it difficult to identify the obstacles these learners face and the supports that could be implemented. Therefore, in this study we use video stimulated recall (VSR), an introspective method, to elicit participants’ thoughts about specific classroom events (Gass & Mackey, 2017; Lyle, 2003). These events relate to the key components of SDT (Ryan & Deci, 2018). Self-determination programs – although implemented outside the classroom setting – have shown positive outcomes for autistic (young) adults, including improvements in goal setting, goal striving, goal attainment, problem-solving skills, executive functioning and emotional regulation (Andrés-Gárriz et al., 2025; Minnaert, 2013; Morán et al., 2021; Oswald et al., 2018). Therefore, understanding how autistic students in the Netherlands and Mexico experience each component of need-supportive teaching may be key to designing classroom-based strategies that enhance autistic adolescents’ enjoyment of lessons and motivation to learn (Block et al., 2022). This is particularly essential for those autistic students who demonstrate lower engagement with classroom tasks (Roorda et al., 2021; Zajic et al., 2020) or those who “struggle with key components of self-determination”, such as goal planning and self-regulating (Block et al., 2022, p. 52).

The main research questions that guided our study were: How do autistic students perceive their experiences with teachers’ provision of autonomy support, structure and involvement? How do they perceive their classroom interactions with teachers and peers?

## Method

### Research Design

In stimulated recall (SR) methods, participants are typically asked to reflect on their thought processes following an event or the completion of a task (Gass & Mackey, 2017). The principle of SR is that they may be able “to relive an original situation with vividness and accuracy if presented with a large number of the cues or stimuli which occurred during the original situation” (Bloom, 1953, p. 161). In SR educational research, videos are the most common type of stimuli used (Zhai et al., 2024), as displaying a video can be more effective in eliciting participants’ thoughts than requesting them to hear audiotapes or read transcripts from the event of interest (Gass & Mackey, 2017). Surprisingly, VSR has not yet been used with autistic students, despite previous research showing that video is highly effective in interventions with autistic children (Delano, 2007). Because VSR is video-based, it may be more appropriate for autistic participants than other research methods, which often fail to build on their strengths, such as being able to process more information at any given time (Remington et al., 2019) or preferring visual stimuli (Harrington et al., 2013). In this regard, Delano (2007) highlighted the need for further research into video-based methods with autistic children. Therefore, we utilized a combination of videos and interview questions (thus, VSR interviews) to “trigger” autistic students’ thoughts about selected segments from two videotaped lessons.

### Participants

Participants were 13 autistic students – six from five schools in the Netherlands and seven from one school in northern Mexico – who took part in a larger (observational) research project that analyzed classroom interactions between teachers and autistic students in mainstream secondary education (Esqueda Villegas et al., 2025). The Dutch secondary education system is structured into three distinct levels: pre-vocational (VMBO), which spans four years; senior general (HAVO), lasting five years; and pre-university (VWO or Gymnasium), which is six years (Ministry of Education Culture and Science, 2007). In Mexico, general secondary education is divided into two tiers: lower and upper secondary education, which include three grades. Students typically enroll in lower secondary education between the ages of 11 and 14, while enrollment in the latter occurs between the ages of 15 and 18 (Gobierno de México, 2022).

To take part in the study, autistic participants required a formal Autism Spectrum Disorder (ASD) diagnosis based on the DSM-5 criteria, provided by a healthcare professional. Co-occurring conditions (e.g., ADHD) were not a reason for exclusion. However, participants needed sufficient communication skills to understand written and visual instructions, which were an essential part of the VSR interviews. They also had to be able to respond to researchers’ questions using spoken, written, or visual language (e.g., by drawing). The researchers collaborated with school coordinators to assess students’ social communication skills and verbal comprehension, ensuring that eligible participants were selected for the study, rather than conducting these assessments themselves. The ratio of male-to-female autistic students in the Dutch sample was 4:2, with an average age of 15 years at the time of data collection. In the Mexican sample, the male-to-female ratio was 6:1, with a mean age of 16.5 years. The characteristics of Dutch and Mexican autistic participants are presented in Tables 1 and 2, respectively.

**Table 1:** Sociodemographic information of Dutch participants

Student pseudonym	Gender	Age at the time of data collection	School pseudonym	School level	School grade	Subject
David	Male	15 years old	School 1	HAVO	4th	Math
Alan	Male	15 years old	School 1	HAVO	4th	Math
Rachel	Female	15 years old	School 2	VMBO	3rd	English
Alex	Male	13 years old	School 3	VMBO	1st	Dutch
Sandra	Female	16 years old	School 4	VWO	4th	Math
Simon	Male	16 years old	School 5	HAVO	4th	Math

Note. VMBO = Pre-vocational secondary education, HAVO = Senior general secondary education, and VWO = Pre-university education

**Table 2:** Sociodemographic information of Mexican participants

Student pseudonym	Gender	Age at the time of data collection	School grade	Subject
Sebastian	Male	17 years old	2nd	Physics
Cesar	Male	17 years old	2nd	Physics
Adrian	Male	16 years old	2nd	Math
Daniel	Male	17 years old	2nd	Math
Alberto	Male	Not disclosed	3rd	Sociology
Jesus	Male	17 years old	3rd	Sociology
Sara	Female	15 years old	1st	English

Note. All students came from the same public school

### Procedure

#### Ethical Approval

for the classroom observations and VSR interviews was granted by the Pedagogical and Educational Sciences ethics review chamber from the Faculty of Behavioural and Social Sciences at the host university (PED-2223-S-0001). Mainstream secondary schools were approached by four (Dutch) research assistants in the Netherlands, while schools in Mexico were contacted by the first author. In each country, a handful of schools were contacted, and while most were open to participating, some either declined due to scheduling constraints or did not respond to follow-up emails. Schools were recruited based on their geographical proximity to the researchers (convenience sampling). School principals and/or coordinators received detailed information about the study. If they expressed interest in participating, information sheets outlining the objectives and procedures were provided to teachers, parents, and the autistic students. It was explicitly indicated that participation would be completely voluntary and no monetary compensation would be given. However, participants were offered a summary of the main findings of the project along with some sweets (if they had no alimentary restrictions).

Written consent was obtained by asking teachers, parents, and autistic students to sign a physical informed consent letter. Parents of other non-autistic students present in class were informed about the study and given the opportunity to object via email to the fact that their child might be captured on camera. None of the parents objected. Once the written consent was obtained from all parties, the next step involved coordinating the video recordings of two lessons with the school director and/or teachers. Around the same time, the four research assistants participated in a three- hour training session covering the theoretical aspects of VSR method. They also conducted hypothetical VSR interviews to familiarize themselves with the general instructions and interview procedure while gaining confidence in asking questions that elicit participants’ thoughts about the events being discussed.

After each lesson was recorded, the first and second author reviewed the footage to identify and select segments to elicit autistic students’ thoughts during the VSR interviews. Notably, although VSR methods allow both researchers and participants to choose the segments for discussion (Gass & Mackey, 2017), in this study, all recall segments were identified by the researchers. This approach was adopted for several reasons. First, prior research has shown that VSR participants can adopt a passive role during interviews, waiting for the researcher to pause the video and ask questions (Nguyen & Tangen, 2017). Second, given that lessons in the Netherlands averaged 46.9 min, while those in Mexico averaged 39.8 min, asking autistic students to watch the entire recordings might have been challenging. For instance, some students on the spectrum struggle to remain engaged for extended periods of time (Zajic et al., 2020), and watching lengthy videos could have led to fatigue. Third, some research methods can be distressing for autistic people, particularly when they do not provide a clear overview on *what* is going to happen next (Lewis, 2009). To minimize distress, the VSR interviews were designed to be as structured as possible. Lastly, it is not unusual in VSR research for researchers to purposefully select the segments for discussion (Nguyen & Tangen, 2017; van der Kleij, 2023).

We purposefully selected video segments for the VSR interviews that related to the different constructs of SDT and classroom interactions that were present during that particular lesson, such as: teacher autonomy support, structure, involvement, individual peer and teacher-student interactions, non-verbal aspects of the lesson, and the student’s own behaviors. Segments for each participant were compiled into a single video. Each segment began with a black screen, indicating whether it occurred during Lesson 1 or 2, along with the corresponding segment number (e.g., Lesson 1, segment 2). After the video was finalized, an individual protocol was developed for each interview, containing general instructions and one or two questions for each segment. Importantly, these questions were not displayed to the participants in the video but were read aloud by a researcher. Table 3 provides examples of the video segments and questions used in (individual) VSR interview protocols.

**Table 3:** Examples of video segments and questions used in VSR interview protocols

Category	Camera perspective	Segment time	Description of segment	Example of Question(s) and Prompt(s)
Teacher: Autonomy support	Teacher	26:20–26:49 (29s)	The teacher explains the homework and gives students the freedom to decide where to start first.	Q: I hear your teacher saying that your homework consists of two parts and that you can decide where you want to start. What did you think then? P: “When my teacher said I could decide where to start my homework, I thought…”
Teacher: Structure	Student	24:17–25:43 (86s)	The teacher is showing a video with an explanation of what an advertisement looks like.	Q: I see your teacher is showing you a video about what an advertisement looks like. What did you think of this? P: “When my teacher showed me a video about what an advertisement looks like, I thought…”
Teacher: Involvement	Student	03:00–03:15 (15s)	The teacher tells the group that they should try to access the online platform today. He says that if they have any problem or can’t access it, they can send him an email.	Q: What did you think when your teacher said you can email him if you have trouble accessing the platform? P: “When my teacher said I could email him if I had any trouble accessing the platform, I thought…”
Teacher – (Autistic) student interaction	Student	43:56–44:46 (50s)	The teacher and the (autistic) student have a one-on-one interaction. The teacher is telling the student step-by-step what he has to type in the calculator.	Q: What were you thinking when your teacher told you step-by-step what you had to do in the calculator? P: “When my teacher told me step-by-step what I had to do in my calculator, I was thinking that…”
Peer interactions	Student	00:00–00:24 (24s)	The student turns to his side so he is now facing two of his classmates. They seem to be discussing the (results of) the task. They have their calculators in their hands.	Q: I see you are discussing the activity with your classmates here. What were you thinking then? P: “When I was discussing the activity with my classmates, I was thinking that…”
Non-verbal aspects of the lesson	Teacher	20:52–21:09 (17s)	Teacher explains the arrows go to the right because it’s a positive force. He uses his hands to point towards the right.	Q: I see your teacher uses his hands to show you that the forces go to the right. What did you think then? P: “When my teacher used his hands to show that the forces go to the right, I was thinking that…”
Student’s behavior	Student	00:33–00:43 (10s)	The student is looking at the whiteboard. He then smiles and seems happy while he solves the task.	Q: What were you thinking when you looked at the whiteboard and smiled? P: “When I looked at the whiteboard and smiled, I thought…”

Once all materials were prepared (which, on average, took 6 days after the last lesson was recorded), VSR interviews were conducted at the preferred location of the autistic student (e.g., school or home). A parent could also be present upon request. The researcher, based on Gass and Mackey’s (2017) VSR research protocol, read the following general instructions to participants:

Hi [student’s name]. How are you doing today? Remember that we videotaped some of your [subject] lessons with a camera? What we’re going to do now is watch a video that has *some parts* of your [subject] lessons and I will ask you some questions about it. I can see what you were doing by looking at the video, but I don’t know what you were thinking. So, what I would like you to do is tell me what you were thinking. You can do this by speaking to me, writing it down on paper, or drawing it. You can decide which option you like better for each question I will ask you. I am going to place the computer between us and I’ll play the video. At some point, I am going to press “pause” on the video and ask you some questions. You can tell me anything that you were thinking at that moment, even if what you were thinking was not related to your [subject] lesson.

Afterwards, the procedure was visually summarized for the student using four cards: (1) First, we watch parts of your lessons together, (2) I press pause on the video, (3) I ask you one or more questions, and (4) you can talk, write or draw what you were thinking at that point in the video. Although the last instruction was adapted to accommodate the diverse communication skills and preferences of autistic people (American Psychiatric Association, 2022), all students in this study opted for verbal responses. In addition, if the student had difficulties understanding the question, the researcher was instructed to instead read it as prompt, for example: “*When my teacher was discussing my test and explaining the planning for the next three weeks*,* I was thinking that…”*. Depending on the student’s response, additional questions were posed to obtain more detailed information. All interviews were audio-recorded with the consent of the participant.

### Data Analysis

The audio recordings from the VSR interviews were transcribed verbatim by Dutch and Spanish native speakers. Next, these were translated to the English language. Thematic analysis was employed to qualitatively analyze and interpret the data (Braun & Clarke, 2022). This process involved researchers’ familiarization with the data through repeated reading of the VSR interview transcripts, assigning initial codes based on SDT, generating themes and sub-themes, and refining and naming the themes to best represent the recall comments made by autistic participants (Braun & Clarke, 2022). It should be noted that data were analyzed using ATLAS.ti 24.0, with both deductive and inductive approaches (Proudfoot, 2023). In the deductive phase, the first and second author initially coded whether the VSR interview questions referred to one of the predefined themes of SDT (autonomy support, structure, and involvement), the non-verbal aspects of the lesson or student’s behaviors. The first reliability check, by calculating percentage of agreement, showed an 85% of agreement between coders. We then further coded the data with an inductive approach, identifying more specific aspects within each SDT theme (e.g., different types of structure, such as “visual supports”), depending on the patterns we found. A second reliability check of this more detailed coding indicated 92% inter-coder agreement. Lastly, the first author applied an open coding to the recall comments of the autistic students and labeled them as “positive”, “neutral”, or “negative”. A discussion took place with the second author if the label was a mixture or seemed unclear.

Since this method is idiosyncratic and an individual protocol was elaborated for each autistic student who participated in this research, no country comparisons were made during the data analysis. Instead, we provide a narrative about the aspects that autistic students recalled having an impact on their learning, particularly in terms of autonomy support, structure, involvement and classroom interactions.

## Results

A total of 181 VSR interview questions were asked to thirteen autistic students from the Netherlands and Mexico, ranging between 11 and 22 questions per participant. The videos used as stimuli lasted from 3:54 to 15:38 min (see Table 4 for further details). Although participants had the possibility of giving written, drawn or verbal responses, all opted for verbal comments. These comments discussed aspects related to the three dimensions of SDT, as well as their interactions with peers. Table 5 illustrates themes and sub-themes and the number of times that positive, neutral or negative comments were made among the autistic students regarding each category.

**Table 4:** Characteristics of the VSR interviews

Student pseudonym	Date of Lessons 1 and 2	Date of VSR interview	Number of segments and questions	Video length (minutes: seconds)
David	March 6 and 10	March 19, 2023	20 segments; 22 questions	15:38
Alan	March 6 and 10	March 19, 2023	16 segments; 18 questions	13:49
Rachel	April 3 and 12	April 17, 2023	10 segments; 11 questions	06:12
Alex	April 5 and 12	April 18, 2023	11 segments; 13 questions	08:06
Sandra	April 12 and 14	April 18, 2023	12 segments; 12 questions	06:32
Simon	June 9 and 12	June 20, 2023	11 segments; 12 questions	12:04
Sebastian	May 8 and 9	May 17, 2023	12 segments, 14 questions	06:23
Cesar	May 9 and 11	May 17, 2023	12 segments; 15 questions	05:49
Adrian	May 8 and 11	May 17, 2023	13 segments; 16 questions	06:13
Daniel	May 8 and 9	May 17, 2023	9 segments; 11 questions	05:12
Alberto	May 9 and 11	May 17, 2023	11 segments; 14 questions	03:54
Jesus	May 9 and 11	May 17, 2023	13 segments; 14 questions	05:34
Sara	May 9 and 11	May 17, 2023	13 segments; 14 questions	07:00

**Table 5:** The appraisal of students’ comments regarding each (Sub)theme

Themes and sub-themes	Negative	Neutral	Positive
Autonomy support	1	-	5
Structure: Discussing previous, current or future lessons	7	7	15
Structure: Guidance throughout the lesson	6	2	15
Structure: Visual supports	-	-	13
Structure: Expected behavior	2	7	5
Structure: Feed-back/forward	5	4	7
Involvement: Being relatable	-	1	6
Involvement: Being understanding, supportive and encouraging	3	5	17
Interactions with teacher	12	12	39
Interactions with peers	1	3	15

Note. Participants could have more than one comment related to a sub-theme

### Theme 1: Autonomy Support

Overall, students received low autonomy support from teachers across lessons and participants. Therefore, only three autistic students were asked about this theme during the VSR interviews. Interestingly, this yielded five positive recall comments and a negative one, as shown in Table 5. For instance, during David’s and Alan’s Math lesson, the teacher gave students the option to change the order of a formula. Both students noted the benefits that this had, such as avoiding getting caught-up with the task or making mistakes:

Huh, is that allowed? Oh yes, that does make it a bit more convenient…It was a helpful comment…Otherwise you got stuck. (David)

I thought it would be useful to know, because there are definitely other questions that would look like this…and could be asked on the test as well. I like that he already gave an example, so that we could not go wrong there. (Alan)

Notably, one student reported that rather than feeling empowered by teacher’s autonomy support, she felt puzzled. In this case, during Sandra’s Math lesson, the teacher said that homework was divided in two parts, and that students could decide where to start. About that moment she recalls: “I found that a bit…confusing, because I prefer a clear assignment where it says: you have to start with this and not free choice to determine that yourself”.

### Theme 2: Structure

Contrary to autonomy-support, teachers did provide high levels of structure to the students. Therefore, all participants were inquired about this matter during VSR interviews. Five sub-themes emerged from the analysis: (1) discussing previous, current and future lessons, (2) guidance throughout the lesson, (3) visual supports, (4) Expected behavior, and (5) feed-back/forward.

#### Subtheme 1: Discussing Previous, Current or Future Lessons

Autistic students generally reported positive experiences when teachers explicitly referred to tasks or topics discussed in previous lessons. These references appeared to enhance autistic students’ workflow and foster their sense of mastery over new material. For instance, David expressed how identifying prior knowledge helped him with the ongoing task:

I thought okay, we’re not starting with paragraph 1 yet. We’ll first get an explanation about what we’ve had in earlier years. And, so that we just repeat that, so that it goes better during the first and second paragraphs…so it was nice.

Similarly, Sebastian noted how recalling successful strategies from another lesson improved his approach to the current one: “In that part [of the lesson] I remembered what I had done in the previous one to see what had worked well for me in that one and repeat it in this one”.

In contrast, some students expressed more neutral responses when teachers outlined ongoing classroom tasks. For example, Rachel felt that these explanations did not stand out in any meaningful way and described them as routine: “It’s just a boring lesson as always, really”.

When teachers explained how the following weeks would unfold, and provided details about upcoming tests, responses varied. Alex, for instance, did not find these “necessary” but several others appreciated it:

I thought it was nice that he provided some overview and that we are also a bit more certain about the material of the test week. Because I hoped we would not get several paragraphs of test material without explanations. So I liked that. (Alan)

I do like the way he brings it [the exam] and that he reminds me a bit about the test. That he also helps think along and I also think that he does that nicely and calmly and not really in a way of: you still have to catch up on this. (Sandra)

Furthermore, one student emphasized that he “Could have forgotten that there was a Math test” (Adrian) if the teacher had not reminded the whole class.

#### Subtheme 2: Guidance Throughout the Lesson

Many autistic students discussed the positive aspects of receiving structured guidance during class. They reported that it helped them to pay more attention to the topic, identify important information, and move forward with their tasks. For example, Simon sought reassurance from his teacher to make sure he was on the right track, recalling how his confidence in task-completion was boosted: “Okay, now that I know it’s okay, I can continue this way”.

Interestingly, while the majority of recall comments were positive about receiving frequent support, two participants mentioned issues that arose with this type of structure, such as difficulties keeping up with the pace of the lesson. Daniel, for instance, struggled to understand an explanation because, as he recalled, “He had never done that [task] on a calculator”. Similarly, for Sebastian, frequent guidance led to over-reliance on the teacher’s scaffolding. He elaborated:

It’s just that sometimes I am used to him [the teacher] telling you exactly what it [the problem] asks you [to do]. Because at least my mom always tells me, like, when my mom gives me an instruction, she is specific. And since the problem does not give you everything specific, it is difficult for me.

These experiences suggest that although step-by-step guidance can be helpful, excessive reliance on this structured support may limit autistic students’ initiative to solve problems on their own and hamper their feelings of being autonomous.

#### Subtheme 3: Visual Supports

Six autistic participants were asked about the visual supports their teachers used during class, such as videos, images or color-written annotations in the whiteboard. All these students recalled only positive experiences regarding these approaches. They particularly emphasized how drawings provided clarity since they were “organized” and made it easier to identify “important information”. The use of videos was also appreciated, as they were often better “explained” and “understood” compared to verbal explanations, as noted by Alex and Simon.

Similarly, Sebastian highlighted that he is a visual learner and always tries “to imagine” things when they are explained to him. Another student, Sara, mentioned her preference for explanations that use different colors. She found it striking, however, that her teacher had never used different marker colors before the recorded lessons. She recalled: “It kind of caught my attention because she had never done that…The truth is that I really liked the teacher’s explanation [with different colors], but it struck me that she had never done it”.

#### Subtheme 4: Expected Behavior

Participants’ reactions were at variance when teachers indicated which behaviors were expected during lessons, such as pay attention or listening. Some experiences were, unfortunately, associated with anxiety. For example, Jesus was frequently called out to focus on the teacher’s explanations. However, the teacher did not understand that his hand-flapping was part of his stimming: “I did hear her talking to me, right? It’s just that sometimes I don’t know how to control…I mean, I am aware of what I’m doing but I don’t realize it”. He also added that he was often distracted by “Voices of classmates”. Other students, such as Rachel, “Did not mind or anything” when the teacher explicitly mentioned her name to re-engage her in the lesson. Similarly, Alberto acknowledged that he would “pay attention in class” after the teacher had ask him to do so.

#### Subtheme 5: Feed-Back/Forward

A recurring theme among autistic participants was their need to be informed about their task performance. Students wanted to identify what they did wrong in order to attain their goals. Adrian shared: “I saw it [the task] again [after the teacher provided feedback]. What I had done. And then I tried to do it again and then the right answer came out”. In addition, students view teachers’ comments as opportunities for reflection:

I wanted to see what had failed me because when I don’t understand things, I like to ask. But I also like to reason to see what failed me or what could I have done wrong. To see what the right process was or why it was the right process. (Sebastian)

While several participants spoke of the benefits of receiving information about *what* they did wrong and *how* they could move forward, many also feared teachers’ addressing their mistakes. For instance, having her mistakes pointed out made Sara feel that she “Should have done better”. Moreover, this often drew the attention of classmates, which negatively impacted the autistic students’ participation:

Many students also laugh when someone does that [make a mistake]. This makes it difficult to even raise that finger to answer, because then, if you then think, I’m not 100% sure and you make a mistake, it’s not fun to be laughed at. (David)

In terms of receiving compliments for their good work, some autistic students did not think much of it. For example, Cesar emphasized that he “Felt almost nothing” when the teacher acknowledged he gave the right answer. Other students, however, were anxious about being complimented, fearing negative reactions from classmates:

I thought for a moment he [the teacher] was going to name me [to compliment him for asking a good question], but luckily that was not the case. I wouldn’t like that very much. People sometimes don’t respond very well to that. (Alan)

### Theme 3: Involvement

Many participants were inquired about their teacher’s involvement during VSR interviews. Consequently, two sub-themes emerged from the analysis: (1) being relatable, and (2) being understanding and supportive.

#### Subtheme 1: Being Relatable

Autistic participants enjoyed when teachers discussed non-academic topics, such as sports. David found it amusing, noting: “I always find it funny to listen to what the teacher is talking about…every lesson it is about something different”. Some students also appreciated teachers who were “funny” or made them laugh with silly voices or personal stories. Rachel particularly enjoyed sharing a special handshake with her teacher, saying: “We actually do that every lesson. So I just like doing that [handshake]”.

#### Subtheme 2: Being Understanding and Supportive

Many autistic participants emphasized their appreciation for teachers who were understanding of their needs and took the time to address their questions in a calm and non-judgmental manner. Sandra’s comments about her Math teacher capture many positive attributes the participants associated with their teachers:

S: I think his classes are actually the best of all the classes I’ve had, so I think he is doing a good job.

Interviewer: Can you explain what that entails?

S: Well, he usually manages the class well… And I like that. And also that he is not judgmental about questions to him. He just wants to explain it until you get it yourself. And that is with some teachers, sometimes they can…they can find it annoying if you don’t understand something they have just explained. And his classes are just very calm and he – when you tell a story he really listens to understand you instead of reacting.

Teachers’ acknowledgement of students’ questions was essential for autistic students to move on with tasks. As Rachel noted, “I thought I had done it [the task] wrong so then she [the teacher] said that I had done it right, so I could just continue”. However, one participant recalled feeling nervous when the teacher checked her progress, saying: “I always get nervous. That feeling. Oh, am I doing it right?”. Similarly, Simon felt unease about making mistakes during teacher-student interactions, even though he knew the teacher would “React calmly”. Other students, like Alex and Daniel, recognized that “It was nice” to know that teachers were available, even when they did not have any questions or needed help at the time.

### Theme 4: Interactions with Peers

Although one participant mentioned that some peers were “Distracting” and interrupted his workflow, many autistic students also acknowledged that peer interactions were helpful for achieving their goals. For example, Cesar had not completely understood the assignment, so he asked a classmate for help. After this interaction, he recalled that he: “Had understood and said ‘Let’s get to work!’”. Similarly, Adrian was struggling to obtain the right answer during independent work, so he decided to discuss the formulas with his classmates:

The formulas were coming out wrong for me, and so I said: Why are they coming out wrong? And then I asked: Hey, how did you guys do it? And they [the classmates] told me that they were doing it in a different way that I wasn’t; that I didn’t see. And then I saw it and said: Ahhh. That’s what it is for.

Notably, some peer-interactions were not related to the learning task at hand. For instance, Alberto and Jesus mentioned that they enjoyed receiving and giving high-fives to classmates. Such interactions, although they are not academically-oriented, may foster a sense of relatedness in the classroom and contribute to the well being among these students.

## Discussion

This study aimed to explore how 13 autistic students from the Netherlands and Mexico perceived their experiences with teachers’ provision of autonomy support, structure, involvement, and classroom interactions. To elicit these students’ thoughts about two video-taped lessons, we employed VSR interviews (Bloom, 1953; Gass & Mackey, 2017). Interestingly, this method not only enabled us to capture their thoughts, but also their emotional reactions (Ahmed et al., 2010; Eynde et al., 2006). Moreover, VSR interviews facilitated a broader exploration of autistic students’ learning experiences, extending beyond the purposefully selected video segments to address events that occurred regularly within the classroom or school context (Meier & Vogt, 2015).

When asked about their experiences with teachers’ provision of autonomy support, autistic students in this study recalled both positive and negative instances. While two participants mentioned that having choices allowed them to move forward and avoid mistakes, one autistic girl reported that it led to confusion about what was expected of her. A possible explanation for these varying responses is that teachers generally provide lower levels of autonomy support (Heyworth et al., 2021), especially compared to structure and involvement (Loopers et al., 2023). As a result, the infrequent opportunities for exercising autonomy may be challenging for autistic learners, who often rely on routines and struggle to cope with uncertainty (Costley et al., 2021). To prevent negative emotions such as stress or anxiety caused by unclear instructions (Esqueda Villegas et al., submitted), it is important for teachers to establish a balance between offering choices and maintaining task structure. Additionally, incorporating more autonomy-supportive practices could help autistic students become accustomed to these approaches in teachers’ repertoires (Shea et al., 2013).

Contrary to autonomy support, all autistic participants were provided with different forms of teacher structure. Overall, these seemed crucial in enhancing their confidence in achieving goals (e.g., completing tasks) and fostering a sense of mastery. Some autistic students, for instance, required building on prior knowledge and connecting it to current lessons to ensure a better workflow. Others valued reminders about upcoming tasks or tests, likely due to concerns about academic performance (Jarman & Rayner, 2015) or executive functioning difficulties (Tamm et al., 2020). Similarly, participants appreciated guidance throughout the lesson. Notably, while step-by-step directions can facilitate autistic students’ problem solving (Anderson et al., 2024) and engage them with their learning, a lack of autonomy might lead autistic students to rely too heavily on their teachers (Shea et al., 2013). Given the limited time teachers often have for one-on-one interactions (Cameron, 2014), they may not always be able to provide the detailed guidance that many autistic students seem to appreciate. Consequently, these students may feel “stuck” without immediate support, underscoring the importance of equipping them in vivo/in-the-moment with the skills to manage tasks independently. Visual supports (which are another component of structure) were unanimously perceived as helpful for organizing and clarifying information. Visual strategies should be utilized more frequently, not only within lessons but also in research methodologies, as it enhances learning and even facilitates behavioral changes by building upon autistic students’ strengths (Delano, 2007; Ravet, 2018).

Another aspect that stood out (in terms of structure) was the process of receiving feed-back/forward. Autistic students not only valued identifying where they went wrong in a task but also appreciated receiving clear next steps to achieve their goals (Hattie & Timperley, 2007; Minnaert, 2013). Notably, many students expressed fear about making mistakes, particularly when teachers addressed these in front of the entire class. Likewise, several participants worried about being praised by teachers, despite SDT highlighting that recognition can fulfill students’ psychological need for competence (Ryan & Deci, 2018). These concerns may stem from autistic people’s heightened fear of being laughed at by others (Keates & Waldock, 2023), especially because both mistakes and praise may trigger negative peer reactions, such as teasing. This fear of being ridiculed, frequently mentioned in VSR interview comments, underscores two key issues. First, feedback and recognition must be delivered in ways that avoid drawing unwanted attention to autistic students. Second, fostering more positive and understanding attitudes among non-autistic peers is crucial to enhancing equitable participation of autistic students in class settings (Settanni et al., 2024).

When teachers in this research demonstrated involvement with their autistic students, many participants appeared to experience a sense of relatedness. They particularly appreciated teachers who engaged in non-academic conversations, displayed a sense of humor, or created personalized handshakes. These findings align with prior research suggesting that approachable, understanding and friendly teachers can enhance feelings of belongingness among autistic students (Baldwin & Costley, 2016; Lebenhagen, 2024). In addition, autistic participants valued teachers who were readily available to answer questions, particularly when they did so in a calm and patient manner. Notably, previous studies have highlighted that some teachers lose patience and express frustration when autistic students struggle to comprehend a topic (Goodall, 2018; Goodall & MacKenzie, 2019; Saggers et al., 2011). Therefore, teachers must recognize that some autistic students may require additional time to process information or further explanations to understand tasks procedures (Esqueda Villegas et al., submitted).

Lastly, all autistic participants identified positive qualities in their interactions with both teachers and classmates. Students mainly relied on these interactions to obtain clarity about tasks and move forward. However, peer-interactions sometimes hindered goal attainment, as some classmates could be distracting and interrupt the autistic students’ workflow. In this line, motivational research highlights that goal accomplishment depends not only on intention but also on the environment, which can hinder task completion and achievement (Minnaert, 2013).

### Implications for Practice

The findings of this study highlight several key implications for secondary school teachers educating autistic students. In line with the literature, autistic participants in this research had varied preferences (Anderson et al., 2024; Saggers et al., 2019). However, they all experienced, to some extent, autonomy, competence, and relatedness when teachers provided autonomy support, structure, and involvement (Snikkers-Mommer et al., 2024). As a result, teachers are encouraged to implement instructional strategies that incorporate all three core components of SDT. For autistic students to truly experience autonomy, it seems crucial that teachers avoid offering unrestricted freedom (during tasks), as it may lead to confusion. Instead, providing alternatives can guide these students toward task completion. As previous investigations suggest, autonomy support and structure can complement one another in practice (Haakma et al., 2017; Jang et al., 2010).

In terms of structure, lessons can begin with a brief recap of previously discussed topics to “refresh” students’ memory and contextualize the new content. Additionally, visual supports can enhance comprehension, as they built on the increased perceptual capacity that many autistic people possess (Remington et al., 2019). Equally valuable is providing timely feed-back/forward, so that autistic learners understand their mistakes and how to proceed. Furthermore, clearly outlining upcoming topics, assignments, and exams can support goal planning, a skill that is often challenging for many autistic people (Block et al., 2022).

In terms of involvement, teachers should demonstrate an understanding of students’ strengths and challenges (Saggers et al., 2011; Sciutto et al., 2012). Additionally, they are encouraged to respond to questions and address mistakes in a non-judgmental manner. Teachers can also incorporate personal anecdotes that resonate with students’ interests and briefly discuss non-academic topics that capture their attention. While it may not be realistic for teachers to adopt a need-supportive teaching style at all times, incorporating these elements into their practice can enhance autistic students engagement (Esqueda Villegas et al., 2025) and further develop their self-determination skills (Block et al., 2022).

### Strengths and Limitations

Notably, our study should be interpreted in light of some limitations. First, the research team purposefully selected the video footage that served as stimuli for the VSR interviews. This means that autistic students could not self-select segments that may have been meaningful for them. Yet, within the purposefully selected video footage, we included a variety of segments in relation to the different constructs of SDT and classroom interactions. This study is the first to explicitly engage autistic students in discussing their teachers’ autonomy-support, structure, and involvement, thereby establishing SDT as a valuable framework for understanding how autistic students can thrive in mainstream education. Second, while the researchers tried to conduct the VSR interviews as immediately as possible after the second lesson took place, this was not feasible at all times due to the complexity of the school context (e.g., public holidays took place in between or scheduling conflicts occurred). Therefore, there is a possibility that some participants provided answers based on the researcher’s expectations rather than their memory of the events (Gass & Mackey, 2017). Nonetheless, our findings show that despite a couple of days taking place between the last lesson videotaped and the VSR interview, the 13 autistic students were able to recall thoughts and emotions about their learning experience. Third, although the voices of autistic students are at the core of this paper, we recognize that autistic people were not directly asked about the design of the study. Since VSR interviews seem a promising methodological strategy to engage autistic people in research and adapt to their individual needs (Harrington et al., 2013), future studies should aim for a participatory research approach in which the autistic community is involved in all stages of the research.

## Conclusion

To the best of our knowledge, this is the first study to use VSR interviews to uncover the thoughts and emotions of autistic students in secondary education. Although examining the full potential of this method in eliciting autistic students’ thoughts and emotions is beyond this paper’s scope, this technique could serve as a solid basis for conducting interviews with this group (Rowe, 2009) and move towards a more inclusive approach in autism research. While VSR is indeed a labor-intensive method, it provided valuable first-hand reflections from participants that could not have been obtained through classroom observations alone (Swit et al., 2024). Additionally, SDT proved to be an effective framework for understanding the needs of this target group in terms of autonomy, competence and relatedness. While we support the notion that “every student with autism is different” (Able et al., 2015, p. 48), our findings highlight fundamental issues that all secondary education teachers should consider in their practice. This need is underscored by Dutch and Mexican secondary education teachers, who have expressed their wish for strategies to support autistic students (Esqueda Villegas et al., submitted), as well as by many teachers worldwide (Ravet, 2018; Saggers et al., 2019; Salceanu, 2020; Sciutto et al., 2012).
